# Effect and Tolerance of N5 and N6 Chemotherapy Cycles in Combination with Dinutuximab Beta in Relapsed High-Risk Neuroblastoma Patients Who Failed at Least One Second-Line Therapy

**DOI:** 10.3390/cancers15133364

**Published:** 2023-06-27

**Authors:** Holger N. Lode, Ruth Ladenstein, Sascha Troschke-Meurer, Linda Struppe, Nikolai Siebert, Maxi Zumpe, Karoline Ehlert, Stefanie Huber, Evgenia Glogova, Patrick Hundsdoerfer, Angelika Eggert, Anna Zaniewska-Tekieli, Walentyna Balwierz, Aleksandra Wieczorek

**Affiliations:** 1 Department of Pediatric Hematology and Oncology, University Medicine Greifswald, 17475 Greifswald, Germany; sascha.troschke-meurer@med.uni-greifswald.de (S.T.-M.); linda.struppe@med.uni-greifswald.de (L.S.); nikolai.siebert@med.uni-greifswald.de (N.S.); maxi.zumpe@med.uni-greifswald.de (M.Z.); karoline.ehlert@med.uni-greifswald.de (K.E.); stefanie.huber@med.uni-greifswald.de (S.H.); 2 Department of Paediatrics, St. Anna Children’s Hospital, Medical University of Vienna, 1090 Vienna, Austria; ruth.ladenstein@ccri.at (R.L.); evgenia.glogova@ccri.at (E.G.); 3 Department for Studies and Statistics and Integrated Research and Project, Children’s Cancer Research Institute (CCRI), St. Anna Kinderkrebsforschung GmbH, 1090 Vienna, Austria; 4 Helios Klinikum Berlin Buch, 13125 Berlin, Germany; patrick.hundsdoerfer@helios-gesundheit.de; 5 Clinic for Pediatric Hematology and Oncology, Charité University Medicine Berlin, 13353 Berlin, Germany; angelika.eggert@charite.de; 6 Department of Pediatric Oncology and Hematology, Jagiellonian University Medical College, 31-008 Krakow, Poland; azaniewska@usdk.pl (A.Z.-T.); balwierz@mp.pl (W.B.); a.wieczorek@uj.edu.pl (A.W.)

**Keywords:** neuroblastoma, relapsed, refractory, chemoimmunotherapy, ganglioside GD2, dinutuximab beta

## Abstract

**Simple Summary:**

Dinutuximab beta is an antibody approved for the maintenance treatment of patients with high-risk neuroblastoma. It is being investigated in combination with different established chemotherapy regimens in various clinical settings. We reviewed the clinical charts of 25 patients with relapsed/refractory neuroblastoma who had failed one or more second-line treatments and were given compassionate use treatment with dinutuximab beta long-term infusion combined with two induction chemotherapy regimens (N5 and N6), recommended by German guidelines. We found no unexpected severe toxicities. Grade 3/4 pain was reported in treatment cycle 1 by four patients, which was reduced to no patients by cycles 3 and 4. Almost half (48%) of patients had a complete, partial or minor response to treatment, despite previous treatment failures. Therefore, combining long-term infusion of dinutuximab beta with these chemotherapy regimens during earlier treatment phases may be beneficial for patients with relapsed/refractory neuroblastoma and should be further evaluated in clinical trials.

**Abstract:**

The anti-disialoganglioside (GD2) monoclonal antibody dinutuximab beta is approved for the maintenance treatment of high-risk neuroblastoma. Dinutuximab beta combined with different chemotherapy regimens is being investigated in various clinical settings. We conducted a retrospective clinical chart review of 25 patients with relapsed/refractory neuroblastoma who had failed ≥1 second-line therapy and received compassionate use treatment with dinutuximab beta long-term infusion combined with the induction chemotherapy regimens N5 (cisplatin, etoposide, vindesine) and N6 (vincristine, dacarbazine, ifosfamide, doxorubicin) recommended by the German Pediatric Oncology and Hematology Group [GPOH] guidelines. The treatment did not result in any unexpected severe toxicities or in any major treatment delays. Grade 3/4 pain was reported by 4/25 patients in cycle 1, decreasing to 0/9 patients in cycles 3 and 4. The median follow-up was 0.6 years. The best response in this group was 48% (12/25 patients), which included three patients with minor responses. At 1 year, the estimated event-free survival was 27% (95% confidence interval [CI] 8–47) and overall survival was 44% (95% CI 24–65). Combining long-term infusion of dinutuximab beta with N5 and N6 chemotherapy demonstrated an acceptable safety profile and encouraging objective response rates in heavily pretreated patients with high-risk neuroblastoma, warranting further evaluation in clinical trials.

## 1. Introduction

Neuroblastoma (NB) is a solid tumor of childhood and approximately 50% of all patients are stratified as high-risk [1], requiring high-intensity multimodal therapies, including chemotherapy, surgery, high-dose chemotherapy following autologous stem cell transplantation combined with iodine-131 metaiodobenzylguanidine (I-131 MIBG) treatment, and radiation therapy [2]. Despite treatment progress over the years, the event-free survival (EFS) rate at 5 years for patients with high-risk NB is below 50% [3,4]. More recently, immunotherapy using monoclonal antibodies against disialoganglioside (GD2) has been introduced in the maintenance phase following multimodal treatment [3]. GD2 is an ideal target due to its abundance on NB cells [5], and loss of GD2 antigens from tumors rarely occurs after receiving antibody therapy [6]. When used in the maintenance phase, the anti-GD2 antibody dinutuximab in combination with interleukin-2 (IL-2) and granulocyte-macrophage colony stimulating factor (GM-CSF), or dinutuximab beta without additional cytokine comedication, increased EFS at 5 years by ~15% [3,7].

An option to further improve outcomes is to include immunotherapy with anti-GD2 antibodies at an earlier phase of the multimodal treatment and combine it with chemotherapy. The first prospective randomized trial exploring chemoimmunotherapy with an anti-GD2 antibody was conducted by the Children’s Oncology Group (COG) in patients with relapsed or refractory NB [8]. Patients treated with dinutuximab plus GM-CSF combined with irinotecan and temozolomide showed an objective response (complete or partial) rate of 41.5% [8]. This combination was also explored in Europe using dinutuximab beta instead of dinutuximab and also omitting GM-CSF in children with relapsed/refractory high-risk NB as part of the BEACON Phase II trial (NCT0230852) [9]. Patients aged 1–21 years with relapsed/refractory high-risk NB with adequate organ function and performance status were randomized in a 1:2 ratio to receive chemotherapy (temozolomide and topotecan) alone or in combination with dinutuximab beta, given concurrently as a 7-day continuous infusion (10 mg/m^2^/day). The study included 65 patients in total. The overall response rates for patients treated with chemoimmunotherapy and those receiving chemotherapy alone were 35% and 18%, respectively (risk ratio 1.66, 80% confidence interval [CI] 0.9–3.06, *p* = 0.19). Progression-free survival rates at 1 year were 57% and 27% for those receiving chemotherapy plus dinutuximab beta and chemotherapy alone, respectively (hazard ratio [HR] 0.63, 95% CI 0.32–1.25, *p* = 0.19). Twelve patients in the chemotherapy-only arm crossed over to receive dinutuximab beta at progression. However, overall survival (OS) did not differ between patients treated with chemotherapy alone and those receiving chemotherapy plus dinutuximab beta (HR 0.99, 95% CI 0.42–2.36; *p* = 0.99). The use of dinutuximab beta in combination with irinotecan and temozolomide in routine clinical practice was also associated with promising objective response rates of 63–64% [10,11]. Based on the positive findings in the relapsed/refractory setting, this chemoimmunotherapy concept was also explored in newly-diagnosed patients with high-risk NB. A single-arm study in the US investigated the combination of the anti-GD2 antibody hu14.18K322A, GM-CSF, IL-2 and the COG induction chemotherapy in newly-diagnosed patients [12]. The objective response rate at the end of induction was 97%, with no patients experiencing progressive disease during induction therapy, suggesting that this chemoimmunotherapy combination may be superior to historical controls [12]. Another single-arm study in the US investigated the combination of dinutuximab plus GM-CSF with the COG induction chemotherapy regimen (cycles 3–5) in newly-diagnosed patients with high-risk NB [13]. The chemoimmunotherapy combination resulted in an objective response of 86.8% at the end of induction, with 11 patients achieving a complete response [13]. However, the use of dinutuximab beta in combination with European NB induction chemotherapy regimens has not yet been evaluated. In preclinical studies, we investigated the effects of dinutuximab beta combined with chemotherapeutics used in European frontline treatment regimens for newly-diagnosed patients with high-risk NB using a NB spheroid model [14]. In the presence of immune cells, the observed cytotoxic effect was up to 17 times stronger in spheroid cultures treated with the chemoimmunotherapy combination compared with controls treated with chemotherapy alone [14]. Based on these preclinical findings and the clinical results observed with the COG induction chemoimmunotherapy combination, it seems plausible to evaluate dinutuximab beta in combination with a European induction chemotherapy in patients with high-risk NB. Since the treatment with anti-GD2 antibodies is associated with the induction of neuropathic pain mediated by GD2 expressed on sensory nerve fibers [15,16], comedication with strong analgesics, including intravenous (IV) morphine, is required to achieve acceptable treatment tolerance [17]. This off-tumor on-target side effect can be ameliorated by administering dinutuximab beta as a long-term continuous infusion [18]. This application method can presumably reduce toxicity and treatment complications during chemoimmunotherapy.

Here, we evaluated the feasibility, tolerance and outcomes of combining dinutuximab beta long-term infusion with the induction chemotherapy regimens N5 and N6, recommended by the German Pediatric Oncology and Hematology Group [GPOH] guidelines [19], in patients with relapsed or refractory NB who had failed at least one second-line therapy.

## 2. Materials and Methods

### 2.1. Patients and Treatment

We performed a retrospective review of the clinical charts of patients with relapsed or refractory high-risk NB, who had received dinutuximab beta immunotherapy combined with the GPOH induction chemotherapy N5 and N6 as part of compassionate use treatment at one of four centers (Berlin, Germany; Greifswald, Germany; Krakow, Poland and Vienna, Austria) between September 2016 and January 2023. All patients had high-risk NB and had experienced a relapse or disease progression following standard induction chemotherapy and had failed to respond to at least one second-line treatment. High-risk NB was defined according to the International Neuroblastoma Staging System (INSS) classification [20,21]. Patients were included if they were aged ≥12 months at diagnosis and had INSS stage 4 NB, or if they had INSS stage 2, 3, 4 or 4S NB with MYCN amplification. Patients with disseminated relapse were also included, regardless of age and stage at diagnosis. In addition, patients had to have measurable or evaluable disease.

Dinutuximab beta combined with GPOH induction chemotherapy N5 or N6 was given to patients for whom other options were not effective. Lack of treatment efficacy was defined as either lack of treatment response (stable disease or continuous progression) or relapse/disease progression following initial response. The treatment patients received for earlier relapses/disease progression was not standardized and was administered according to the institution’s standard of care.

Chemoimmunotherapy cycles consisted of sequential applications of N5 (40 mg/m²/day cisplatin continuous infusion over 96 h, 100 mg/m²/day etoposide continuous infusion over 96 h, vindesine 3 mg/m² on day 1 IV over 1 h) and N6 (vincristine 1.5 mg/m²/day on days 1 and 8 IV over 1 h, dacarbazine 200 mg/m²/day on days 1–5 over 1 h, ifosfamide 1.5 g/m²/day continuous infusion over 115 h, doxorubicin 30 mg/m²/day on days 6 and 7 IV over 4 h) both in combination with dinutuximab beta (10 mg/m^2^/day, continuous infusion for 5 days; cumulative dose 50 mg/m^2^/cycle) [19]. Dinutuximab beta treatment started on day 5 of the respective chemotherapy cycle. This starting point was selected to reduce the risk of capillary leak syndrome induced by dinutuximab beta during hyperhydration with 3 L/m^2^/24 h of fluid required for the safe application of the N5/N6 chemotherapy cycles [19]. The planned treatment interval between cycles was 28 days depending on hematologic recovery from previous cycles. Granulocyte colony stimulating factor (5 µg/kg/day) was given to all patients starting on day 9 of each cycle to support granulocyte recovery, which is standard for this type of chemotherapy. Four treatment cycles of dinutuximab beta combined with chemotherapy were planned (two cycles with N5 and two with N6) in anticipation of reduced chemotherapy treatment tolerance in such a heavily pretreated patient population, but the definitive number of cycles depended on disease progression and tolerability. Unacceptable toxicity was defined as any unexpected grade 3 or 4 adverse events (AEs) that did not improve to grade 1 or 2 events before the start of the next treatment cycle, or grade 4 hematologic events that did not improve between cycles. Comedication for dinutuximab beta included IV morphine (cycle 1: 30 µg/kg/hour, as long as needed, and from cycle 2 as needed); oral gabapentin (all cycles: 10 mg/kg/day on day 3; 2 × 10 mg/kg/day on day 4; 3 × 10 mg/kg/day on days 5–10 [or longer, if needed]) and metamizole (all cycles: 80 mg/kg/day continuously on days 5–10 [or longer, if needed]).

### 2.2. Assessments

Tumor response was evaluated at baseline and after two and four cycles of dinutuximab beta plus N5 and N6 chemotherapy, and at any time when progression/relapse was suspected. Response was assessed according to the most recent International Neuroblastoma Response Criteria [22] and included minor responses. The evaluation was performed locally during a tumor board meeting of oncologists, surgeons, and radiologists. The disease status was assessed using computed tomography and/or magnetic resonance imaging, scintigraphy with I-123 or I-131 MIBG and bone marrow investigation using anti-GD2 immunocytochemistry. Positron emission tomography was carried out for patients with MIBG non-avid disease. 

AEs were recorded and graded using the Common Terminology Criteria for Adverse Events (CTCAE) version 4.3. Pain was evaluated on a scale from 0 to 10 (the Wong-Baker FACES Pain Rating Scale), with 0 indicating no pain and 10 the worst imaginable pain [23]. The use of IV morphine as a surrogate parameter of pain was also determined on each day of each cycle. 

The concentration of dinutuximab beta in the serum of treated patients was assessed by a validated enzyme-linked immunosorbent assay (ELISA) method as previously described [24].

### 2.3. Statistical Analyses

The data cut-off for the analyses was 28 February 2023. Survival curves were generated using the Kaplan–Meier method and compared statistically using a log-rank test (*p* < 0.05 was considered statistically significant) [25]. Patients were censored at the date of their last assessment. OS was defined as the time from the start of chemoimmunotherapy until death from any cause. To analyze paired data for IV morphine use with missing values over time, we used a mixed-effects model for repeated-measures ANOVA. Multiple comparisons against cycle 1 were conducted with a Dunnett’s *post-hoc* test. Statistical analysis was performed using GraphPad Prism version 9.5.1 for Windows (GraphPad Software, San Diego, CA, USA, www.graphpad.com, accessed on 1 April 2023).

## 3. Results

### 3.1. Patient Characteristics

Twenty-five patients with relapsed or refractory NB who had failed at least one second-line treatment received sequential N5 and N6 chemotherapy combined with dinutuximab beta. Patient baseline demographics and disease characteristics are shown in Table 1, and the details of first-line and second-line treatment are in Supplementary Table S1. The median age at diagnosis was 3.1 years (range 0.05–8.72; 95% CI 2.5–4.3) and 10 (40%) patients had tumors with MYCN amplification. The majority of patients (92%) had metastatic disease at diagnosis. Most patients (15/25, 60%) had received first-line treatment according to the HR-NBL International Society of Paediatric Oncology European Neuroblastoma (SIOPEN) protocol (rapid COJEC: cisplatin [C], vincristine [O], carboplatin [J], etoposide [E], and cyclophosphamide [C]) [26] and nine (36%) patients had received the NB2004 GPOH protocol [27] that consists of N5 and N6 chemotherapy cycles. One patient with localized stage 3 NB was treated according to the Low and Intermediate Risk Neuroblastoma European Study (LINES) SIOPEN protocol (ClinicalTrials.gov identifier: NCT01728155; group 10, etoposide/carboplatin and carboplatin/doxorubicin) with an initial response before the patient developed a disseminated relapse. All patients developed a relapse or progression of their disease and experienced a lack of efficacy following at least one second-line therapy. Approximately half of the patients (12/25; 48%) had a first, 11/25 (44%) a second and 2/25 (8%) a third relapse or progression (Table 1). Nine patients (36%) had previous therapy with dinutuximab beta. The median time from diagnosis to first relapse or progression was 1.2 years (range 0.05–5.80; 95% CI 1.0–2.2). All 25 patients received at least one cycle, 17 received two, and nine received three and four cycles of dinutuximab beta combined with N5/N6 chemotherapy (60 cycles in total). The reason for treatment discontinuation was progressive disease.

### 3.2. Safety

During the 60 evaluable cycles, there were no unexpected severe toxicities, and there was no increase in severe non-hematologic toxicity from cycle to cycle, except for grade 3/4 infections (Table 2). The high frequency of grade 3/4 hematologic toxicity as well as grade 3/4 infections were expected in this heavily pretreated cohort. Inflammatory grade 3/4 AEs related to dinutuximab beta, such as allergic or anaphylactic reactions and cytokine release syndrome, were not observed. Only one patient developed grade 3 hypotension in cycle 1, which did not reoccur in subsequent cycles. Pain related to dinutuximab beta was highest in cycle 1 (grade 3/4: 4/25 patients; 16.0%) and decreased to 0% in cycles 3 and 4 (Table 2).

**Figure 1:**
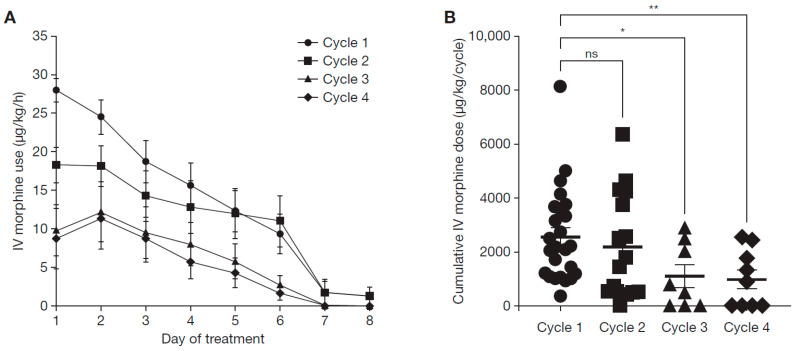
Use of intravenous morphine. The daily use of IV morphine within cycles in µg/kg/h (**A**) and the cumulative IV morphine dose in µg/kg/cycle (**B**) were analyzed. The differences between cycle 1 and cycles 3 and 4 were statistically significant (Dunnett’s *post-hoc* test; * *p* = 0.0104, ** *p* = 0.0020). IV, intravenous; ns, not significant.

Similarly, the daily use of IV morphine decreased within cycles (Figure 1A) and the cumulative IV morphine dose significantly decreased in later cycles (Figure 1B), a phenomenon also observed when dinutuximab beta is given without chemotherapy [28]. We also determined the time periods between each cycle for each patient to evaluate if there were major delays compared with the planned interval of 28 days. The median time period from day 1 cycle 1 to cycle 2 was 28 days (95% CI 25.6–34.0), cycle 2 to 3 was 35 days (95% CI 32.6–40.0) and cycle 3 to cycle 4 was 34 days (95% CI 27.0–37.2).

### 3.3. Tumor Response and Survival Analyses

Patients were assessed after two and four planned chemoimmunotherapy cycles, and the response was analyzed with and without considering minor responders (Table 3). The best response was 48% (12/25 patients), where three responding patients had a minor response, and 36% (9/25 patients) without the inclusion of minor responders. 

**Figure 2:**
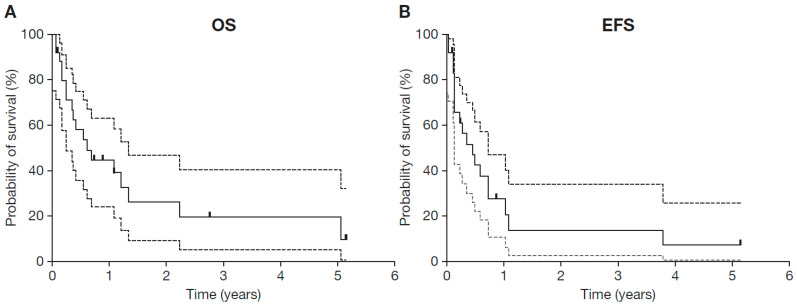
Kaplan-Meier estimates for (**A**) overall survival and (**B**) event-free survival. Grey shaded curves indicate the upper and lower range of the 95% CIs. EFS probability at 1 year was 27% (95% CI 8–47) and the OS probability at 1 year was 44% (95% CI 24–65). Ticks indicate censored patients. CI, confidence inverval; EFS, event-free survival; OS, overall survival.

The median follow-up was 0.6 years, with an EFS probability at 1 year of 27% (95% CI 8–47) and an OS probability at 1 year of 44% (95% CI 24–65) (Figure 2). Patients with MYCN-amplified NB showed a similar probability for 1-year OS and a tendency towards a decreased 1-year EFS probability compared with patients without MYCN-amplified NB (OS: 42% [95% CI 8–76] versus 43% [95% CI 17–69], *p* = 0.396; EFS: 0% [95% CI 0.0–0.0] versus 38% [95% CI 0.12–0.65], *p* = 0.162).

**Figure 3:**
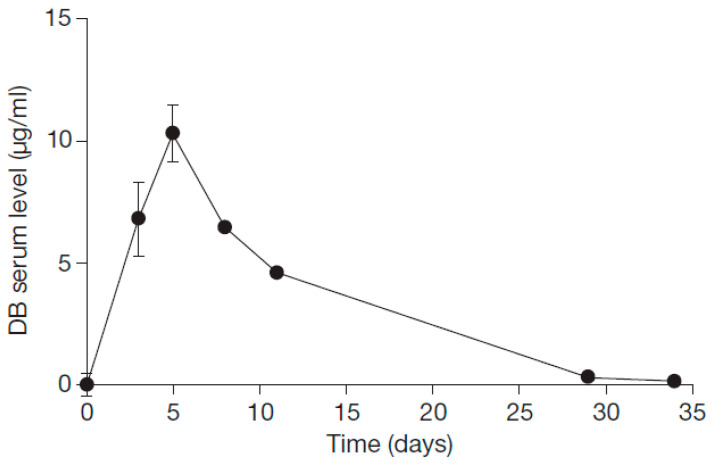
Serum concentration time curves of dinutuximab beta. The concentration of dinutuximab beta was determined before, during and after 5-day continuous infusion of 50 mg/m^2^ from days 1–5 and followed until the treatment start with the antibody in cycle 2 at indicated time points. Data represent the mean value ± standard error of the mean. Error bars not visible are covered by the symbol. DB, dinutuximab beta.

We monitored the dinutuximab beta serum levels in eight patients with available samples (Figure 3). We observed an end-of-infusion concentration of 10.30 ± 1.49 µg/mL and a trough concentration of 0.14 ± 0.11 µg/mL.

## 4. Discussion

The use of dinutuximab beta is established for the treatment of high-risk NB as maintenance or post-consolidation therapy for newly-diagnosed patients [3,29] and for patients with a history of relapsed or refractory NB, with or without residual disease [28,30,31]. In both treatment scenarios, dinutuximab beta is applied after chemotherapy. However, monoclonal antibodies developed for the treatment of patients with solid tumors were also successfully used and approved in combination with chemotherapy, such as trastuzumab for breast cancer [32,33] and cetuximab for colorectal carcinoma [34,35]. In the US, combination regimens comprising anti-GD2 immunotherapy and induction chemotherapy recommended by the COG have produced encouraging survival outcomes in patients with newly-diagnosed high-risk NB [12,13]. It is, therefore, reasonable to explore dinutuximab beta in combination with European induction chemotherapy regimens for the treatment of patients with high-risk NB. The current European high-risk NB protocol (HR-NBL2 SIOPEN) comprises an ongoing prospective randomization comparing rapid COJEC with N5/N6 induction chemotherapy in newly-diagnosed patients. In addition, a Phase I clinical trial to explore the treatment tolerance to dinutuximab beta in combination with rapid COJEC and N5/N6 chemotherapy in newly-diagnosed patients with high-risk NB is currently being set up.

We have shown in preclinical studies that dinutuximab beta works synergistically with chemotherapeutics used in frontline induction chemotherapy regimens for NB [14]. The synergistic effect seems primarily attributed to the combined toxicity of antibody-dependent cellular cytotoxicity and chemotherapy, probably due to an increased stress ligand expression induced by the chemotherapeutic compound that renders NB cells more susceptible to immunological attacks [14]. There are also reports of a direct cytotoxic effect of anti-GD2 antibody 14G2a in vitro, which contains the same variable region as dinutuximab beta. The antibody demonstrated a dose-dependent cytotoxic effect against IMR-32 human NB cells [36]. The induced cell death was thought to be of apoptotic nature based on observed apoptotic characteristics, such as cleavage of caspase 3, a notable increase in caspase activity, and an increase in Annexin V- and propidium iodide-positive cells. Interestingly, the anti-GD2 antibody was found to exert a synergistic effect with doxorubicin and topotecan, and an additive effect with carboplatin in causing cell death in vitro [36], but the underlying mechanism is not entirely understood. A preclinical study has also shown that the anti-GD2 antibody 14G2a downregulates PI3K/Akt/mTOR signaling network in human NB cell lines (IMR-32, CHP-134, LA-N-1 cells), which is a critical pathway for growth and proliferation of NB cells [37].

In light of the increasing interest to evaluate the addition of dinutuximab beta in combination with frontline induction chemotherapy regimens, we retrospectively analyzed the feasibility and effect of adding dinutuximab beta to N5 and N6 chemotherapy cycles in relapsed and refractory NB patients, which are core elements of the induction chemotherapy regimen recommended by the GPOH guidelines to treat newly-diagnosed patients with high-risk NB [19], in patients with relapsed or refractory NB. We found that the addition of dinutuximab beta as a 5-day continuous infusion (10 mg/m^2^/day, 50 mg/m^2^/cycle) is feasible without any unexpected severe toxicities and without major delays to sequentially apply the combination. The severe toxicities that were observed in our cohort reflect those previously reported for both dinutuximab beta and the N5 and N6 GPOH induction chemotherapy regimens when administered for the treatment of high-risk NB separately [27,38]. In line with previous studies evaluating dinutuximab beta as a long-term infusion [28,39], immunotherapy-related pain was reported most frequently in the first treatment cycle and occurred less often in subsequent cycles, with no patients experiencing grade 3/4 pain during cycles 3 or 4. This reduced frequency of pain over time was also reflected in daily morphine use, which decreased over the course of the study. Interestingly, no patients in our study had grade 3/4 cytokine release syndrome or allergic or anaphylactic reactions, all of which are typically reported in patients treated with dinutuximab beta [29,38]. In addition to an encouraging toxicity profile, the feasibility of this regimen is further supported by the relatively consistent time intervals between planned treatment cycles. The administration of subsequent cycles was not substantially delayed from the planned 28 days in our cohort, and a maximum median interval of 35 days was recorded, limiting disruption to the planned treatment regimen. 

The drug concentrations achieved were in an expected range and well above an effective level of 1 µg/mL at the end of the antibody infusion. We also observed a best overall response rate of 48% (including minor responders) and 36% (excluding minor responders, i.e., only patients with complete and partial responses). These findings are similar to the overall response rate of 35% reported for the BEACON trial [9] and lower compared with combinations of dinutuximab beta with irinotecan and temozolomide, with reported objective response rates of 63% [10] and 64% [11] in patients with relapsed/refractory NB. However, the group of patients reported here had a higher risk profile and was more heavily pretreated. Our encouraging findings also suggest that combining dinutuximab beta with induction chemotherapy regimens may also be a possible treatment option during induction therapy in the first-line setting of patients with high-risk NB. It may also be possible to administer dinutuximab beta even earlier during the chemotherapy cycle.

## 5. Conclusions

We report the feasibility of combining dinutuximab beta with the N5 and N6 chemotherapy cycles of the GPOH induction chemotherapy regimen, with an acceptable safety profile and encouraging objective clinical response rates in heavily pretreated patients with relapsed/refractory high-risk NB. Our findings encourage further evaluation of this chemoimmunotherapy approach in clinical trials.

## Figures and Tables

**Table 1:** Patient demographics and disease characteristics.

Category	Patients (*N* = 25)
Age at diagnosis	
Mean, years	3.4
Median (range), years	3.1 (0.05–8.72)
<18 months	3
18 months–5 years	18
>5 years	4
Sex, *n*	
Male	19
Female	6
INSS stage	
4	22
4s *	1
3 ^§^	2
2	0
1	0
MYCN	
Amplified	10
Non-amplified	14
Unknown	1
Time to 1st relapse/progression	
Mean, years	1.9
Median (range), years	1.2 (0.05–5.80)
Type of relapse	
Primary tumor site alone	1
New site	1
Skeleton alone	1
Combined (primary site, skeleton and new site)	22
Number of relapses/progressions	
1	25
2	11
3	2

Data are number of patients unless stated otherwise. * Patient presented with disseminated relapse 9 months after initial diagnosis; ^§^ One patient was MYCN-amplified, and both developed a disseminated combined relapse 3 years and 1.5 years after initial diagnosis, respectively. INSS, International Neuroblastoma Staging System.

**Table 2:** Toxicity of N5 and N6 chemotherapy combined with dinutuximab beta.

Adverse Event	Cycle 1		Cycle 2		Cycle 3		Cycle 4	
Grade	1/2 *n*/*N* (%)	3/4 *n*/*N* (%)	1/2 *n*/*N* (%)	3/4 *n*/*N* (%)	1/2 *n*/*N* (%)	3/4 *n*/*N* (%)	1/2 *n*/*N* (%)	3/4 *n*/*N* (%)
Cardinal toxicities								
Pain	10/25 (40.0)	4/25 (16.0)	5/15 (33.3)	2/15 (13.3)	5/9 (55.6)	0/9 (0)	3/9 (33.3)	0/9 (0)
General condition	18/25 (72.0)	5/25 (20.0)	14/17 (82.4)	2/17 (11.8)	6/9 (66.7)	0/9 (0)	7/9 (77.8)	1/9 (11.1)
Fever	15/25 (60.0)	2/25 (8.0)	14/17 (82.4)	0/17 (0)	5/9 (55.6)	1/9 (11.1)	6/9 (66.7)	1/9 (11.1)
Allergic reactions	0/25 (0)	0/25 (0)	2/17 (11.8)	0/17 (0)	1/9 (11.1)	0/9 (0)	1/9 (11.1)	0/9 (0)
Capillary leak syndrome	3/25 (12.0)	1/25 (4.0)	1/16 (6.3)	0/16 (0)	0/9 (0)	0/9 (0)	0/9 (0)	0/9 (0)
Cytokine release syndrome	2/23 (8.7)	0/23 (0)	0/15 (0)	0/15 (0)	0/8 (0)	0/8 (0)	0/8 (0)	0/8 (0)
Hypotension	1/25 (4.0)	1/25 (4.0)	2/17 (11.8)	0/17 (0)	2/9 (22.2)	0/9 (0)	3/9 (33.3)	0/9 (0)
Neurologic toxicity								
Central neurotoxicity	0/24 (0)	1/24 (4.2)	0/17 (0)	0/17 (0)	0/8 (0)	0/8 (0)	0/8 (0)	0/8 (0)
Peripheral neurotoxicity	0/25 (0)	0/25 (0)	1/16 (6.3)	0/16 (0)	1/8 (12.5)	0/8 (0)	0/8 (0)	0/8 (0)
Hematologic toxicity								
Decreased hemoglobin	6/25 (24.0)	19/25 (76.0)	1/17 (5.9)	16/17 (94.1)	0/9 (0)	9/9 (100)	3/9 (33.3)	6/9 (66.7)
Decreased white blood cell count	2/25 (8.0)	23/25 (92.0)	0/17 (0)	17/17 (100)	0/9 (0)	9/9 (100)	0/9 (0)	9/9 (100)
Decreased granulocytes	1/25 (4.0)	24/25 (96.0)	0/17 (0)	17/17 (100)	0/9 (0)	9/9 (100)	0/9 (0)	9/9 (100)
Decreased platelets	1/25 (4.0)	24/25 (96.0)	1/17 (5.9)	16/17 (94.1)	1/9 (11.1)	8/9 (88.9)	0/9 (0)	9/9 (100)
Gastrointestinal toxicity								
Nausea/vomiting	12/24 (50.0)	0/25 (0)	4/17 (23.5)	0/17 (0)	2/9 (22.2)	0/9 (0)	1/9 (11.1)	0/9 (0)
Diarrhea	14/24 (58.3)	0/24 (0)	5/17 (29.4)	1/17 (5.9)	3/9 (33.3)	0/9 (0)	3/9 (33.3)	0/9 (0)
Constipation	12/24 (50.0)	1/24 (4.2)	10/17 (58.8)	0/17 (0)	5/9 (55.6)	0/9 (0)	3/9 (33)	0/9 (0)
Stomatitis	4/24 (16.7)	0/24 (0)	4/17 (23.5)	1/17 (5.9)	2/9 (22.2)	0/9 (0)	3/8 (37.5)	0/8 (0)
Cardiac toxicity								
Cardiac function	0/17 (0)	1 */17 (5.9)	0/10 (0)	0/10 (0)	0/4 (0)	0/4 (0)	0/4 (0)	0/4 (0)
Cardiac ischemia/infarction	0/17 (0)	0/17 (0)	0/10 (0)	0/10 (0)	0/4 (0)	0/4 (0)	0/4 (0)	0/4 (0)
Cardiac arrythmia	0/17 (0)	0/17 (0)	0/10 (0)	0/10 (0)	0/4 (0)	0/4 (0)	0/4 (0)	0/4 (0)
Myocarditis	1/22 (4.5)	0/22 (0)	0/14 (0)	0/14 (0)	0/8 (0)	0/8 (0)	0/8 (0)	0/8 (0)
Hypertension	2/25 (8.0)	2/25 (8.0)	1/17 (5.9)	1/17 (5.9)	0/9 (0)	0/9 (0)	1/9 (11.1)	0/9 (0)
Renal toxicity								
Increased creatinine	3/25 (12.0)	2/25 (8.0)	3/17 (17.6)	0/17 (0)	2/9 (22.2)	0/9 (0)	2/9 (22.2)	0/9 (0)
Proteinuria	4/18 (22.2)	0/18 (0)	2/12 (16.7)	0/12 (0)	0/5 (0)	0/5 (0)	0/3 (0)	0/3 (0)
Hematuria	4/20 (20.0)	1/20 (5.0)	3/14 (21.4)	0/14 (0)	0/5 (0)	0/5 (0)	0/4 (0)	0/4 (0)
Decreased glomerular filtration rate	2/18 (11.1)	1/18 (5.6)	1/13 (7.7)	0/13 (0)	1/5 (20.0)	0/5 (0)	2/5 (40.0)	0/5 (0)
Infections								
	10/25 (40.0)	3/25 (12.0)	5/17 (29.4)	3/17 (17.6)	4/9 (44.4)	2/9 (22.2)	1/8 (12.5)	3/8 (37.5)

* Grade 4 cardiac function toxicity was due to sepsis.

**Table 3:** Treatment response in 25 evaluable patients.

	Treatment Response		
Patient No.	2 Cycles	4 Cycles	Best Response
01	PD		PD
02	PD		PD
03	PD		PD
04	SD	PD	SD
05	SD		SD
06	SD	SD	SD
07	PD		PD
08	PR		PR
09	PD		PD
10	PR	CR	CR
11	PR	CR	CR
12	PR	PR	PR
13	MR	PR	PR
14	MR	MR	MR
15	MR	MR	MR
16	PR		PR
17	PD		PD
18	PR		PR
19	PD		PD
20	MR	MR	MR
21	CR		CR
22	PD		PD
23	PR		PR
24	PD		PD
25	SD		SD
Total MR	4	3	3
Total PR	7	2	6
Total CR	1	2	3
PR/CR, *n*/*N* (%)	8/25 (32)	4/25 (16)	9/25 (36)
MR/PR/CR, *n*/*N* (%)	12/25 (48)	7/25 (28)	12/25 (48)

CR, complete response; PR, partial response; MR, minor response; SD, stable disease/no response; PD, progressive disease.

## Data Availability

The data presented in this study are available on request from the corresponding author.

## Supplementary Materials

The following supporting information can be downloaded at: https://www.mdpi.com/article/10.3390/cancers15133364/s1, Table S1: Treatment history.
