# Supplementary material for: Effect and Tolerance of N5 and N6 Chemotherapy Cycles in Combination with Dinutuximab Beta in Relapsed High-Risk Neuroblastoma Patients Who Failed at Least One Second-Line Therapy
Source: Cancers (Basel). 2023 Jun 27;15(13):3364. doi: 10.3390/cancers15133364 (PMC10341209; doi:10.3390/cancers15133364)
Supplement: Supplementary file 1 [file cancers-15-03364-s001.zip › cancers-2445598-supplementary.pdf]

Table S1: Treatment history

Patient No.	First-line treatment	Second-line treatments		
		**1st relapse/ progression**	**2nd relapse/ progression**	**3rd relapse/ progression**
01	NB2004 GPOH	Ceretinib	-	-
02	NB2004 GPOH	TEC	ICE	-
03	NB2004 GPOH	RIST	Ceretinib	-
04	NB2004 GPOH	Ceretinib	-	-
05	NB2004 GPOH	Ceretinib	Melphalan	-
06	HR-NBL-1 SIOPEN	I/T	PACE	Haplo/DB
07	HR-NBL-1 SIOPEN	ICE	-	-
08	NB2004 GPOH	TVD	-	-
09	HR-NBL-1 SIOPEN	I/T	T/C	-
10	NB2004 GPOH	T/C	ICE	-
11	NB2004 GPOH	I/T	-	-
12	HR-NBL-1 SIOPEN	T/C	ICE	-
13	NB2004 GPOH	I/T	-	-
14	HR-NBL-1 SIOPEN	T/C	-	-
15	HR-NBL-1 SIOPEN	T/T	DB + IL-2	-
16	HR-NBL-1 SIOPEN	I/T + bev	-	-
17	LINES SIOPEN (Group 10)	I/T + DB	-	-
18	HR-NBL-1 SIOPEN	TOTEM	-	-
19	HR-NBL-1 SIOPEN	I/T	-	-
20	HR-NBL-1 SIOPEN	I/T	DB + IL-2	-
21	HR-NBL-1 SIOPEN	CEVAIE	I/T	DB
22	HR-NBL-1 SIOPEN	I/T	-	-
23	HR-NBL-1 SIOPEN	I/T + bev	-	-
24	HR-NBL-1 SIOPEN	I/T	I/T + DB	-
25	HR-NBL-1 SIOPEN	TVD	I/T + DB	-
Total	25	25	12	2

For NB2004 GPOH and HR-NBL-1 SIOPEN frontline induction chemotherapy refer to Berthold F, *et al. Ann Oncol.* 2020;31(3):422−429 and Garaventa A, *et al. J Clin Oncol*. 2021;39(23):2552−2563, respectively. For haplo/DB refer to Flaadt T, *et al. J Clin Oncol*. 2023. doi: 10.1200/JCO.22.01630. bev, bevacizumab; CEVAIE, carboplatin, epirubicin, vincristine, ifosfamide, actinomycin D, etoposide; DB, dinutuximab beta; ICE, ifosfamide, cyclophosphamide, etoposide; IL-2, interleukin-2; I/T, irinotecan/temozolomide; PACE, cisplatin, doxorubicin, cyclophosphamide, etoposide; T/C, topotecan/cyclophosphamide; TEC, topotecan, etoposide, cyclophosphamide; TOTEM, topotecan, temozolomide; T/T, topotecan/temozolomide; TVD, topotecan, vincristine, doxorubicin; RIST, rapamycin, irinotecan, dasatinib, temozolomide.
